# Proactively Delivered Digital Mental Health Support for Health Care Workers: Usability and Acceptability Evaluation

**DOI:** 10.2196/74086

**Published:** 2025-12-09

**Authors:** Lauren Southwick, Rachel Gonzales, Lisa Bellini, David A Asch, Nandita Mitra, Mohan Balachandran, Courtney Benjamin Wolk, Emily M Becker-Haimes, Rachel Kishton, Sarah Beck, Raina M Merchant, Anisk K Agarwal

**Affiliations:** 1 Department of Emergency Medicine Perelman School of Medicine University of Pennsylvania Philadelphia, PA United States; 2 Center for Health Care Transformation and Innovation Penn Medicine Philadelphia, PA United States; 3 Department of Medicine Perelman School of Medicine University of Pennsylvania Philadelphia, PA United States; 4 Department of Biostatistics, Epidemiology, and Informatics Perelman School of Medicine University of Pennsylvania Philadelphia, PA United States; 5 Department of Psychiatry Perelman School of Medicine University of Pennsylvania Philadelphia, PA United States

**Keywords:** well-being, health care workforce, digital intervention, feasibility, COVID-19 pandemic, usability, acceptability

## Abstract

**Background:**

Health systems are investing in mental health and well-being support tools and resources for health care workers (HCW). Considering the mental health strain facing HCWs, there is a need to optimize the current mental health delivery model.

**Objective:**

This study aimed to evaluate the usability and acceptability of a proactive digital mental health approach (Cobalt+;Penn Medicine), which included services proactively sent to HCWs via text messaging, including (1) monthly automated text messaging reminders and links to Cobalt, and (2) bimonthly text-message–based measures of depression and anxiety.

**Methods:**

This study used the System Usability Scale (SUS), Net Promoter Score (NPS), and open-ended questions to capture Cobalt+ participants who received proactive digital mental health tools and resources. Descriptive summary statistics were used for SUS and NPS outcome measures, and a chi-square test was used to detect group differences. Open-ended questions were analyzed using a qualitative open coding process by 2 coders. Research team members calculated interrater agreement (Cohen κ above 0.80).

**Results:**

A total of 162 of 642 HCWs randomized to Cobalt+ (25.2%) visited Cobalt due to a proactive text message and completed usability and acceptability measures. The mean age was 38.9 years, most were female (90.7%), 56.8% White, 53.1% married or partnered, and 34.6% engaged in shift work. The mean SUS score was 74.43 (median score 72.5). Participants said they mostly “browsed” the online mental health platform. Cobalt+ received an NPS of 13.7. When asked to elaborate on their experience, 2 categories (eg, positive and negative experiences) with 13 subcategories were identified. Most participants noted the brief process that helped prioritize mental health: “Forget otherwise. Puts in forefront of my mind,” and “Your texts do remind me to take stock of my current feelings.”

**Conclusions:**

A proactive digital mental health approach may help overcome barriers in the uptake of services that are otherwise passively available to HCWs. This study demonstrated that the proactive approach is generally usable, modestly acceptable, and further supplemented by HCW feedback. These findings suggest the approach’s viability and the need for additional research toward improvement and broader implementation.

**Trial Registration:**

ClinicalTrials.gov NCT05028075; https://clinicaltrials.gov/study/NCT05028075

## Introduction

Health care workers’ (HCWs) well-being is essential to ensure high-quality patient care and the durability of the workforce [1]. The rates of stress and burnout among HCWs were high before the COVID-19 pandemic, and these rates have persisted or increased [2]. The pandemic resulted in acute stressors directly impacting an already strained health care system. HCWs faced unique challenges during the pandemic related to rapid shifts in care, moral injury, and concerns about risks of infection [3-5].

In response to the demands of the pandemic, health systems have invested in mental health and well-being tools, including in-person resources such as peer-to-peer networks and digital mental health programs that offer remote or asynchronous mental health care and resources to their employees [2,6-9]. Digital mental health programs have garnered attention as they offer privacy and confidentiality to overcome stigma and can be scaled to employee bases [10]. These digital platforms offer a variety of resources such as individual health assessments, resilience tools, journaling prompts, progress visualization, and connection to mental health resources [9,11]. Research has begun to explore the utility of digital platform use among diverse US employers. Bondar et al [10] found that among 66 US employers across 40 states from 2018 to 2021 (of which 5% were included health care organizations), 1132 employees participated in an employee-sponsored digital program. They found among their sample of employees with moderate anxiety or depression and who attended at least 1 mental health appointment had improved depression and anxiety symptoms [10]. These results are promising, but a gap exists in exploring how digital platforms were leveraged specifically among HCWs.

Health systems have created their own digital platforms and tools to support HCW well-being and mental health [5,12,13]. The Mount Sinai Health System launched the Wellness Hub app [12] which includes self-administered surveys, resilience tools, journaling prompts and well-being videos, and personalization features. Understanding of the tool use was measured by metrics such as the number of downloads, surveys submitted, and engagement metrics (eg, number of openings). The US Department of Veterans Affairs created the COVID Coach app [9], which included tools and resources to support well-being, track personal growth, and visualize progress over time. Penn Medicine created Cobalt, an open-source online centralized suite of mental health resources designed to support HCWs through a stepped care model [12]. Cobalt offers depression and anxiety screening and connects users with a well-being coach (for mild symptoms), a care manager (for moderate symptoms), or a clinician (for more severe symptoms). In 2020 Cobalt had over 10,000 users, 200,000 page views, 1400 one-on-one appointments, and over 1000 group sessions [12].

There is a need to optimize the current mental health delivery model for HCWs as mental health strains persist in medicine. Tong et al [14] notes a need to shift from focusing solely on symptom alleviation but toward proactive individual health-seeking and health-promoting behaviors [15]. For example, the current and usual care model requires HCWs to personally initiate multiple steps (1) identify a personal need for support; (2) know where to seek support and care; (3) download the app, visit a website, or schedule an appointment; and (4) use the resource or attend the appointment. In this context, the HCW has to seek the resources they need, and there may be several barriers to completing each step. This is particularly relevant, as mental health conditions can compromise insight, motivation, and decision-making, thereby making self-directed engagement in care more challenging.

To address this gap, this study team conducted a randomized controlled trial (RCT) among 1275 HCWs at a large, urban, academic health system that demonstrated reduced depression and anxiety symptoms after 6 months of a proactive digital approach to Cobalt [16]. Despite these encouraging findings, little is known about the HCW experience receiving proactive resources. Although they may overcome barriers to accessing tools, some may view receiving proactive mental health services as off-putting, intrusive, and counterproductive. Research is needed to better understand the HCW experience of workplace mental health programs and proactive approaches.

This analysis aims to evaluate the usability and acceptability of Cobalt+ (Penn Medicine), a proactive and digital mental health approach, and capture the participant. Two key objectives guided the analyses: (1) evaluate usability and acceptability through widely used and validated measures; (2) capture participants’ perceptions and experience through open-ended questions. We hypothesize that participants will find this approach both usable and acceptable. As digital mental health programs continue to scale across institutions, it is critical to better understand how participants experienced and engaged with the intervention, as these insights can directly inform the ongoing efforts to support HCW mental health.

## Methods

### Overview

This is a subgroup analysis of the Cobalt+ RCT, a separately reported RCT [16]. The parent RCT randomized 1275 HCWs to test the effect of proactive digital mental health messaging (Cobalt+). A total of 633 of the HCWs were randomized to usual care, and 642 HCWs were randomized to Cobalt+. Eligible HCWs were employed by a large, urban, academic health system in the Northeast and provided informed consent. Participants were excluded if they were non–English speaking, did not have daily access to a smartphone, or did not participate in at least 4 hours per week of patient care. Recruitment was conducted via email from January to May 2022. Eligible and consenting individuals were enrolled and randomized on a rolling basis, and the final participant completed follow-up in March 2023 [16].

Cobalt+ intervention participants received a suite of services via proactive text messaging, including (1) monthly automated text messaging reminders and links to Cobalt, and (2) bimonthly text-message–based measures of depression and anxiety. Participants included in this analysis are those randomized to Cobalt+ and who visited the Cobalt platform due to the text messages and completed usability and acceptability measures. The trial was approved by the institutional review board at the University of Pennsylvania (IRB protocol #: 848844) and preregistered on ClinicalTrials.gov (identifier: NCT05028075).

### Data Collection

The intervention and data collection process was entirely remote. Outcome measures and text messages were administered in English and electronically using a Health Insurance Portability and Accountability Act (HIPAA)–compliant research platform to facilitate remote engagement for clinical trials and care [17].

### Outcome Measures and Data Sources

All participants completed baseline demographics before randomization; variables included age, sex, race, ethnicity, marital status, and professional role. They also completed depression (Patient Health Questionnaire-9 [PHQ-9] [18]) and anxiety assessments (Generalized Anxiety Disorder-7 [GAD-7] [19]), both of which are widely used and are validated instruments and have been used in multiple studies and efficiently screen for symptoms of depression and anxiety. All participants completed PHQ-9 and GAD-7 follow-up assessments at months 6 and 9. Usability and acceptability outcome measures for this analysis were collected cross-sectionally at the 6-month survey only. The datasets generated or analyzed during this study are not publicly available due to participant privacy, but a deidentified dataset is available from the corresponding author on reasonable request. Of note, the PHQ-9 and GAD-7 data are available for the public to access through the National Institute of Mental Health Data Archive, which makes data available across studies from a variety of scientific domains.

#### Usability

Usability was measured using the System Usability Scale (SUS), a 10-item validated survey [20]. The SUS is a widely used measure of perceived usability across digital products and platforms. We selected SUS because it is brief, simple to administer electronically, and extensively used to evaluate digital products. It has been used to evaluate HCW experience of novel, innovative tools [21]. SUS is calculated by scoring the 10 responses on a 5-point Likert scale. Odd-numbered items’ scores are subtracted by 1, while even-numbered items are subtracted by subtracting the response from 5. Then the total score is multiplied by 2.5 to yield a final usability score. SUS produces a score ranging from 0 to 100, with higher scores indicating better usability. A SUS score above 68 is typically considered above average. Open-ended questions captured how Cobalt+ participants used the platform. We used the SUS scores and open-ended questions to measure Cobalt’s usability among study participants.

#### Acceptability

Acceptability was measured by the Net Promoter Score (NPS), a common assessment to capture user loyalty and satisfaction [22] NPS was selected for its brevity, interpretability, and widespread use in evaluating new products or services. NPS is often used to evaluate health systems or clinical programs and is a common metric for acceptability partly due to its sensitivity to detractors [21,23]. NPS measures the likelihood of recommending the program to a friend or colleague. It creates a score by subtracting the “detractors” (scores of 0-6) from “promoters” (scores of 9-10). A score of 7 or 8 is considered neutral. For instance, to calculate NPS, you calculate the proportion of promoters minus the proportion of detractors. NPS can range from –100 to 100; scores above 20 indicate “good” customer satisfaction, while scores above 50 represent “strong” customer satisfaction [22]. Open-ended responses assessed general satisfaction and experience with Cobalt+.

### Analyses

Descriptive summary statistics were used for usability (SUS) and acceptability (NPS) outcome measures, and a chi-square test was used to detect group differences (Stata SE 17, Stata Corp LLC). All available data were analyzed; no additional procedures for handling missingness were undertaken.

Open-ended questions were analyzed using a qualitative open coding process and a general inductive approach. Two trained coders (MS and LS) jointly read 50 responses and drafted the outline of the codebook together. The 2 coders self-identified as female, and one as White and one as South Asian. Each coder had 10+ years of experience conducting and analyzing qualitative data. Each coder then independently read 50 more responses to adjust the codebook as needed. The coders met in person every 100 responses to resolve discrepancies and finalize coding procedures. If consensus was not met during these sessions, a senior research team member and qualitative researcher (AA) adjudicated. Coding decisions and codebook revisions were documented in an audit trail to enhance transparency and rigor. Following adjudication of any areas of discrepancy, the coders shared an additional 10 responses to review coding for interrater reliability and overall comprehensiveness. Following adjudication of any areas of discrepancy, coders independently coded the remaining responses. The coders used Microsoft Excel (Version 16.91; Microsoft Corp) to calculate interrater agreement (Cohen κ above 0.80). Strategies used to ensure reliability and validity in the qualitative data included a comprehensive audit trail, checks between coders, and biweekly team debriefings.

### Ethical Considerations

The trial was approved by the institutional review board at the University of Pennsylvania (IRB protocol #: 848844), preregistered on ClinicalTrials.gov (Identifier: NCT05028075). Written informed consent was obtained from all participants. A safety protocol was approved by the institutional review board and overseen by a 3-member independent data and safety monitoring board. Participants reporting suicidal ideation through surveys or text messages were promptly contacted by the University Employee Assistance Program (EAP) for a safety assessment, with up to three outreach attempts within 3 days. Automated safety messages with crisis resources were triggered by predetermined self-harm–related keywords. All participants received information about national suicide prevention resources and mental health support at study completion. Participants received compensation for completing the baseline survey ($50), 6-month survey ($100), and 9-month survey ($50) via electronic gift card. The study was conducted in accordance with the Declaration of Helsinki and local regulations. Participant data were deidentified and stored on encrypted servers.

## Results

### Overview

Of the 642 HCWs randomized to the intervention. Of the 642 Cobalt+ participants, 25.2% (162/642) visited the Cobalt platform due to the proactive text messaging, completed usability and acceptability outcomes, and were included in this analysis. Among the 162 participants who completed the outcomes, the mean age was 38.9 (SD 10.5) years, with nearly half (48.2%) aged 18-35 years. The majority were female (147/162, 90.7%), 56.8% White (92/162), 53.1% (86/162) married or living with a partner and approximately one-third engaged in shift work (56/162, 34.6%). In terms of professional roles, 27.8% were nurses, 12.4% were physicians or advanced practice providers, and 25.9% were in managerial positions. Baseline mental health symptom scores were mild, with mean PHQ-9 and GAD-7 scores of 6.5 (SD 5.3) and 6.4 (SD 4.9), respectively. There were no significant differences in terms of demographics and baseline mental health measures (eg, PHQ-9 and GAD-7) between those who completed the assessments and the overall study sample, except for gender (χ²_640_=8.86; *P*=.00), with a higher proportion of female participants and managers (χ²_640_=6.58; *P*=.04) who completed the outcome measures (Multimedia Appendix 1).

### Evaluation Outcomes

#### Usability

**Figure 1:**
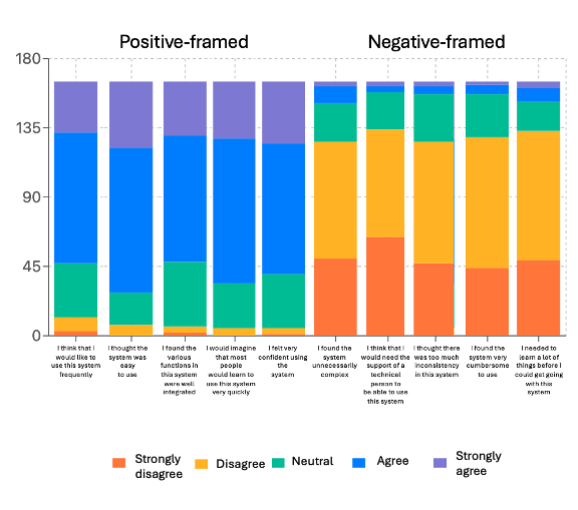
System Usability Scale responses.

The mean SUS score was 74.43 (median score 72.5). Many respondents strongly agreed or agreed to “I felt very confident using the system,” (125, 75.7%) and strongly agreed or agreed to “I think that I would like to use this system frequently,” (118, 71.5%). Most participants (222, 81%) strongly disagreed or disagreed with “I think that I would need the support of a technical person to be able to use this system,” (134, 81.2%). Despite Figure 1’s supplemental insights, validated usability rests on the total SUS score rather than individual usability domains.

#### Qualitative Feedback

Participants who visited Cobalt due to a Cobalt+ text message most visited Cobalt’s videos, podcasts, and articles (95/162, 58%; Table 1).

**Table 1:** Cobalt+ resources are the most used.

Resource type	Frequency, n (%)
Videos, podcasts, and articles	95 (58.3)
Group virtual well-being session	5 (3.1)
Individual coaching session	23 (14.1)
Appointment with a mental health professional	30 (18.4)
Other	10 (6.1)

While the open-ended comments suggest a range of uses. A few respondents explicitly stated that they did not use any resources. Some participants passively browsed out of curiosity or preliminary interest, such as “didn't use anything, just browsed,” and “seeing what options are available if needed.”

#### Acceptability

Among 162 respondents, the majority of respondents were “promoters” 45.7% (74/162, 45.9%), 22% were “neutral” (36/162, 22.2%), and 32% were “detractors” (52/162, 32.1%); which equated to an NPS of 13.7. This NPS score and respondent breakdown suggest “good” acceptability.

#### Qualitative Feedback

Two categories (eg, positive and negative experiences) with 13 subcategories were identified through open-ended questions asking participants to elaborate on their satisfaction and experience receiving the proactive text messages (Table 2).

**Table 2:** Categories, subcategories, and illustrative quotes.

Categories and subcategories		Frequency, n (%)	Illustrative Quote
**Positive experience**			
	Mental health	36 (16.3)	It was a very easy way to be reminded to check in on myself. Your text do remind me to take stock of my current feelings
	Easy	35 (15.8)	This tool is very useful and easy to access Text messages make it easier in busy times
	Positive sentiment	23 (10.4)	I like that they reach out to me, and offer suggestions I did not mind receiving the text with updated information and suggestions.
	Care	19 (8.6)	Nice to know there is someone there It feels like someone understands and is thinking of you Was not an overbearing amount of messages just reminders that you were there for me.
	Helpful	15 (6.8)	I like that they reach out to me, and offer suggestions The reminders were helpful for me to take time to care for myself. Helpful reminders that I can look at on my own terms
	Brief	9 (4.1)	They are concise and to the point. Brief messages, nice to be checked on : )
**Negative experience**			
	Negative sentiment	18 (8.1)	Too many texts I do not find any benefit from the text messages. They just feel like spam. Did not find it helpful
	Not applicable	16 (7.2)	I do not always see my text in a timely mater Little time to schedule yet more appointments
	Did not use	13 (5.9)	Did not feel the need I already see a therapist
	Ignored	12 (5.4)	Most of the time when I received text messages I just look at them real quick and then easily forget about them.
	Intrusive	7 (3.2)	They were helpful reminders, but felt like it was in the way and obstructive to my day Just intrusive, deal with it right away or it disappears. Rarely able to stop and deal with it. Text messages also do not come to my laptop or iPad and the site is easier to access from there
	Overwhelming	3 (1.4)	I have been so overwhelmed with everything else that I did not really take the time to read them because it seemed cumbersome

The positive experiences included general reflections on how participants felt about Cobalt+ components. Within this, subcategories emerged around messages being brief (19/162, 4%), feeling cared for (22/162, 5%), the importance of mental health support (49/162, 11%), and their preference for text message outreach (52/162, 11%). Most participants noted that the brief engagement helped prioritize their mental health. A participant said, “Your texts remind me to take stock of my current feelings.” Several participants appreciated the convenience of receiving messages directly on their phones and valued the freedom to engage with them at their own pace. One participant shared that it “was not an overbearing amount of messages, just reminders that you were there for me,” while another shared, “It was nice being reached out to and checked on.” Participants highlighted the study’s benefits, such as “I did not mind receiving the text with updated information and suggestions.” Overall, participants found the messages easy to engage with (98/642, 21%); a participant said, “Text messages make it easier in busy times,” and they were often helpful, especially when delivering reminders.

The negative experiences captured critiques and challenges associated with the proactive approach. Some participants reported ignoring or not using the messages altogether, feeling that they did not align with their needs or were intrusive. A participant shared, “Most of the time when I received text messages, I just look at them real quick and then easily forget about them.” Others felt the texts were “too many” or that they were “helpful reminders but [were] in the way and obstructive to my day.” Furthermore, a small minority found them to be “cumbersome” because they were “so overwhelmed with everything else that I didn't really take the time to read” (Table 2).

## Discussion

### Principal Results

The results of the primary RCT demonstrated improved mental health outcomes among those HCWs randomized to Cobalt+, a proactive service sent via text messaging, including (1) monthly automated text messaging reminders and links to Cobalt, and (2) bimonthly text-message-based measures of depression and anxiety [16] This analysis investigates how usable and acceptable the proactive support approach was to HCWs and offers 2 main findings. First, Cobalt+ had “good” usability (SUS score=74.43). Previous literature notes that an SUS score above 68 is above average, while scores between 70 and 84 are considered “good usability” [20]. In addition to the SUS composite score, descriptive statistics for individual SUS items were provided to illustrate response patterns across usability domains. When analyzing open-ended usability feedback, the most used resources were videos, podcasts, and articles. This is an important finding as videos, podcasts, and articles are largely cost-effective and can reach a broad audience simultaneously. Second, the NPS score (n=13.7) was positive and, in a range, considered to reflect moderate to low loyalty, suggesting both promise and opportunity for improvement [22]. Despite the positive NPS score, it is essential to note that our sample had a substantial proportion of detractors (52, 32%) and this proportion should not be overlooked.

Open-ended questions provided additional context about opportunities to improve the proactive approach. Lack of time, not needing therapeutic services, or having their own mental health provider were often expressed as the main reasons for not accessing resources. However, some participants enjoyed the proactive outreach and wrap-around support. This finding speaks to the value of the proactive approach where the support and resources are centered around the user. This is particularly relevant, as many digital platforms require users to seek out resources, and mental health conditions can compromise insight, motivation, and decision-making, thereby making self-directed engagement in care. This analysis highlights the importance of “quick and easy” survey assessments and how these periodic check-ins “allow [participants] the freedom to answer when it is convenient… reminds [them] when it's time to self-reflect” and how the frequent outreach made participants feel “cared for.” Yet, a smaller group found the text messaging to be intrusive. Future research could allow individuals to customize their communication type (ie, text message or email) and how often they receive notifications. Yet, this customization does not overlook implementation challenges (eg, integration across systems, resource allocation, and clinical support) [14,24], structural considerations (eg, disparities in access to digital tools), and cultural concerns (eg, stigma and trust in employer-sponsored tools outside of a research context) [25]; however, it is a first step to personalized mental health and well-being outreach, which will lay the foundation for future iterations, refinements, and adaptations to meet the HCWs’ mental health and well-being needs [26].

The digital strategy to send resources and assessments to HCWs was overall found to have good usability and was modestly received. This study reveals formative and important preliminary data that remote text message engagement can extend the reach of mental health platforms for HCWs. Proactive outreach to intermittently engage, assess, and connect health care workers allows one to prioritize mental health and well-being and motivate them using existing resources [27]. This approach also maintains privacy and provides a unique strategy at scale, which can be tailored to the needs of an individual over time.

### Comparison With Prior Work

This is among the first large mental health–focused RCTs to collect participants’ feedback and feasibility metrics among HCWs during the pandemic and beyond. Prior research found a positive impact of an employer-sponsored behavioral health program on improved depression and anxiety symptoms through increased psychotherapy sessions [28,29]. This is a noteworthy difference compared to the Cobalt+ proactive approach and study sample for 2 reasons. First, Maeng et al [28] analyzed individuals whose baseline scores were more than or equal to 10 on depression (PHQ-9) or anxiety (GAD-7) validated measures [28], whereas the Cobalt+ sample had mild baseline PHQ-9 and GAD-7 scores (Multimedia Appendix 1). Given that Cobalt+’s sample exhibited mild baseline symptoms, there was limited potential for measurable improvement in depression and anxiety. Second, despite the Cobalt+’s samples’ mild baseline scores, nearly 20% used appointments with a mental health professional (Table 1). This suggests that individuals with subthreshold forms of depression and anxiety also seek out mental health professional support.

Among digital platforms catered to HCWs, our analysis is most similar to the COVID Coach [9] and Wellness Hub [11] apps. The COVID Coach, smartphone-based app, was tested through an RCT among 40 South African physicians. It was noted to have adequate feasibility acceptability as measured by the SUS and client satisfaction questionnaire (CSQ-8). COVID Coach’s SUS score was slightly higher than Cobalt+’s with 76.6 (SD 14.6). Our analysis did not use the CSQ-8 since Cobalt+ provided a variety of tools and resources and was not limited to mental health services. Nevertheless, the authors recommend future in-depth qualitative interviews to capture users’ experience with the app and patterns of use [9]. In its design and implementation, Cobalt+ also closely parallels the Wellness Hub app [11], sharing similarities in its resource offering and being deployed within a large health system in the Northeast United States. However, to our knowledge, Wellness Hub has not been extensively evaluated. Golden et al [11], reports back-end metrics such as the number of HCWs downloaded the app, the total number of baseline surveys submitted, the number of users submitting additional surveys postbaseline, and general app engagement metrics (eg, the number of app openings). Similarly, their evaluation is lacking the user experience of the app [11].

### Limitations

This analysis has several limitations. First, not all study participants (24.6%) completed the month 6 assessment, and fewer completed the outcome measures (n=162). This limits our generalizability in reflecting the larger participant experience due to the limited responses. Usability and acceptability findings should be interpreted carefully due to this response bias. Second, study participants were recruited from one large, urban health system, and second, the inclusion criteria required participants to have daily access to a smartphone; this criterion may limit generalizability, especially within the context of the known racial and ethnic “digital divide.” This divide may be smaller in employed health care workers, and smartphone ownership continues to expand in the United States [30]. Third, a higher proportion of female participants completed the outcome than the overall study sample measures (Multimedia Appendix 1). Interpretation of our findings and external validity may be limited for dissimilar HCW samples. Fourth, study participants were HCWs willing to complete 2 online surveys; therefore, selection and nonresponder bias may be present. Fifth, participants who opted into the study may have experienced greater distress and were likelier to prefer mobile interventions than those who declined participation. Sixth, validated survey measures lack some specification. For instance, the SUS provides a subjective, global measure of usability and unfortunately does not specify specific domains such as navigation, error prevention, and efficiency. Similarly, despite the NPS’s widespread use, the acceptability thresholds are derived mainly from commercial rather than health care contexts [22]. Our analysis reported SUS and NPS scores and open-ended feedback separately. Seventh, there remains stigma associated with accessing mental health care, which may also contribute to selection bias and limited generalizability. Rather than integrating mental health records or treatments, we relied on self-reported data from participants and data on appointments made. Self-report measures have limitations due to social desirability and recall errors [31,32]. Further research is needed to continuously seek feedback from those using the program to stay relevant and responsive to ongoing and changing stressors.

### Conclusions

Proactive mental health services may help overcome expected barriers to uptake of passively available services. This study demonstrated that Cobalt+, a proactive digital mental health program using proactive text messaging, is modestly usable and acceptable based on two key evaluation metrics, further supplemented by HCW feedback. These findings suggest the program's viability and the need for additional research toward improvement and broader implementation. In this study, we report on the insights gained using simple, scalable text messaging to reach a large group of diverse HCWs. Future studies are needed to test this novel approach across clinical settings and professional roles and in large health systems.

## Appendix

Multimedia Appendix 1 Supplementary material.

## Abbreviations

CSQ-8: client satisfaction questionnaire-8
GAD-7: Generalized Anxiety Disorder-7
HCW: health care worker
HIPAA: Health Insurance Portability and Accountability Act
NPS: Net Promoter Score
PHQ-9: Patient Health Questionnaire-9
RCT: randomized controlled trial
SUS: System Usability Scale
