# Supplementary material for: Proactively Delivered Digital Mental Health Support for Health Care Workers: Usability and Acceptability Evaluation
Source: JMIR Form Res. 2025 Dec 9;9:e74086. doi: 10.2196/74086 (PMC12728399; doi:10.2196/74086)
Supplement: Multimedia Appendix 1 [file formative_v9i1e74086_app1.docx]

Online Supplement

eTable 1. Demographic and professional characteristics

| **Characteristics** | **Study Sample** | All Cobalt+ Participants (n = 642) | | |
| --- | --- | --- | --- | --- |
|  |  | **Did not complete outcome** | **Completed Outcome** | **P-value** |
|  | **(n=642)** | **(n=480)** | **(n=162)** |  |
| Age, mean(std) [range] | 41.5 (10.6) [21 – 74] | 41.5 (10.7) [21 – 74] | 41.8 (10.6) [25 – 72] | 0.728 |
| Age group, n(%) |  |  |  | 0.627 |
| *18-35* | 296 (46.1) | 218 (45.4) | 78 (48.2) |  |
| *36-50* | 249 (38.8) | 192 (40.0) | 57 (35.2) |  |
| *51-64* | 86 (13.4) | 63 (13.1) | 23 (14.2) |  |
| *>=65* | 11 (1.7) | 7 (1.5) | 4 (2.5) |  |
| Female, n(%) | 534 (83.2) | 387  (80.6) | 147  (90.7) | **0.003** |
| Race, n(%) |  |  |  | 0.099 |
| *Asian* | 55 (8.6) | 44 (9.2) | 11 (6.8) |  |
| *Black* | 160 (24.9) | 108 (22.5) | 52 (32.1) |  |
| *Other* | 28 (4.4) | 21 (4.4) | 7 (4.3) |  |
| *White* | 399 (62.1) | 307 (64.0) | 92 (56.8) |  |
| Hispanic, n(%) | 39 (6.1) | 30 (6.3) | 9 (5.6) | 0.749 |
| Married or with partner, n(%) | 367 (57.2) | 281 (58.4) | 86 (53.1) | 0.225 |
| Shiftwork, n(%) | 267 (41.6) | 211 (44.0) | 56 (34.6) | 0.109 |
| Manager, n(%) | 153 (23.8) | 111 (23.1) | 42 (25.9) | 0.037 |
| Profession Role, n(%) |  |  |  | 0.226 |
| *Physician & advance practice providers* | 89 (13.9) | 69 (14.4) | 20 (12.4) |  |
| *Nurse* | 206 (32.1) | 161 (33.5) | 45 (27.8) |  |
| *Other* | 347 (54.0) | 250 (52.1) | 97 (59.9) |  |
| Baseline mental health symptom characteristic |  |  |  |  |
| Depression (PHQ-9), mean (std) [range] | 5.9 (5.2)  [0 – 25] | 5.7 (5.1)  [0 – 25] | 6.5 (5.3)  [0 – 24] | 0.111 |
| Anxiety (GAD-7), mean (std) [range] | 6.0 (4.9)  [0 – 21] | 5.9 (4.9)  [0 – 21] | 6.4 (4.9)  [0 – 20] | 0.275 |
